# An Ethnopharmacological, Phytochemical and Pharmacological Review on Lignans from Mexican *Bursera* spp.

**DOI:** 10.3390/molecules23081976

**Published:** 2018-08-08

**Authors:** Maria Carla Marcotullio, Massimo Curini, Judith X. Becerra

**Affiliations:** Department of Pharmaceutical Sciences, University of Perugia, via del Liceo, 1-06123 Perugia, Italy; massimo.curini@unipg.it (M.C.); jxb@email.arizona.edu (J.X.B.)

**Keywords:** *Bursera*, Burseraceae, lignans

## Abstract

The genus *Bursera* belongs to the family Burseraceae and has been used in traditional Mexican medicine for treating various pathophysiological disorders. The most representative phytochemicals isolated from this genus are terpenoids and lignans. Lignans are phenolic metabolites known for their antioxidant, apoptotic, anti-cancer, anti-inflammatory, anti-bacterial, anti-viral, anti-fungal, and anti-protozoal properties. Though the genus includes more than 100 species, we have attempted to summarize the biological activities of the 34 lignans isolated from selected Mexican *Bursera* plants.

## 1. Introduction

The genus *Bursera* Jacq. ex L. (family Burseraceae, order Sapindales), named after the Danish botanist Joachim Burser (1583–1639), is a monophyletic genus [1] that includes about 105 species of small trees and shrubs distributed from Southern U.S. to Peru and the Caribbean, particularly in Mexico (ca. 92 species) [2]. These plants are characterized by the production of resins that are exuded from the trunk and leaves and provide a chemical defense against specialized herbivores [3]. Once dried, the resin obtained by *Bursera* spp. is called “copal”, a term which is also used to describe a large group of resins characterized by hardness and a relative high melting point, which also is found in other plants [4]. The loss of the essential oils and the oxidation and polymerization processes transform copal into amber.

The phytochemistry of this genus is characterized by the presence of volatile metabolites such as simple hydrocarbons and terpenoids as well as phenolics [5,6,7,8]. Among the compounds present in the volatile fraction, heptane, α- and β-pinene, β-phellandrene, and limonene are among the most frequent [5], whereas β-caryophyllene and germacrene D are the most common sesquiterpenes in the genus *Bursera* [8]. Cembrane and verticillane diterpenoids are often present [9,10,11]. Pentacyclic triterpenoids are largely present in the resin of several species, and the study of triterpenoidic composition of resins is important to define the botanical origin of archaeological samples of copal [12]. In *Bursera microphylla* resin, malabaricane triterpenoids were also found [11]. Leaves and branches of some *Bursera* also contain flavonoids [13,14,15] and luteolin 3′-*O*-rhamnoside is very common [13].

Lignans are naturally occurring plant phenolics, biosynthetically derived from phenylpropanoids, that are important components in foods and medicines; their chemical and biological properties have been reviewed [16]. The aim of this review is to summarize literature findings on the botanical characterization, distribution, ethnopharmacology, and biological activities of Mexican *Bursera* that produce lignans. Different *Bursera* species have been sorted according to, and synonyms are those reported in, the Plant List Database [17]. Unless otherwise specified, common names are those reported by Lemos and Rivera [18]. The phytochemistry was analyzed by data reported in the SciFinder database. Images of the species reported in this paper can be found on the Enciclovida web site [19].

## 2. Genus *Bursera*

Members of this genus are typically small- to medium-sized trees or shrubs, mostly dioecious, succulent, and with resin canals in vascularized tissues. Their leaves are deciduous, imparipinnate, or sometimes unifoliolate or trifoliolate (occasionally bipinnate). Their flowers are small, almost always unisexual, three- to six-merous. Their fruit is a dehiscent two- to three-valve drupe with a fleshy to coriaceous skin, and with a pyrene (the stone or pit that contains the seed), cartilaginous to bony, enveloped totally or partially by an arillate structure.

The taxonomy of the genus *Bursera* is based on morphological characteristics of fruit, bark, and leaves, as well as molecular data. Currently, there are two recognized subgenera: one subgenus named *Bursera* (previously called section Bursera) that includes species commonly known with the general vernacular name of “cuajiotes”, and the other is called *Elaphrium* (previously called section Bullockia) that comprises species with the general common name of “copales” [20,21,22]. The most conspicuous difference between the subgenera is the bark: in subgenus *Bursera*, it tends to be colorful and exfoliating, whereas in *Bursera* subgenus *Elaphrium,* it is likely to be complete (not exfoliating), and grey or reddish grey. However, although bark helps in species identification because it is easy to see, whether the bark is complete or exfoliating is not an absolute difference between the two groups. Setting the bark aside, the most reliable distinction between the two subgenera is the number of locules in the ovary (three in subg. *Bursera* vs. two in *Elaphrium*), and the number of valves in the fruit (three in subg. *Bursera* vs. two in *Elaphrium*) [20,21]. Another distinguishing trait is the presence of well-developed cataphylls (small bract-like leaves that appear before “true leaves” and are short-lived) in subgenus *Elaphrium* and absent or very inconspicuous in subgenus *Bursera* [23]. Toledo further divided section *Bursera* into three groups that can be distinguished by the color of the exfoliating bark: mulatos, red cuajiotes, and yellow cuajiotes [24]. Furthermore, the section *Bullockia* was divided into two groups: pseudoaril-covered fruits group and partially covered fruits group [24]. In 1980, Gillet changed the name of section *Bullockia* into *Elaphrium* due to the fact that some characteristics of this section resemble those of *Elaphrium tomentosum* Jacq. [25]. Phylogeny studies by Becerra and Venable allowed the recognition of four different groups in section *Bursera*: the *simaruba* group (massive trees, trilobate cotyledons, red exfoliating bark, poor producing resin (*mulatos*)), the *microphylla* group (medium-sized trees or shrubs, multilobate cotyledons, yellow to red exfoliating bark, highly resinous), the *fagaroides* group (medium-sized trees or shrubs, multilobate cotyledons, highly resinous), and the *fragilis* group (medium-sized trees, multilobate cotyledons, red exfoliating bark, highly resinous (*cuajiotes*)) [22]; and two groups in *Elaphrium*: *copallifera* (seed completely or at least two-thirds covered by pseudoaril) and *glabrifolia* (seed partially covered or at least less than two-thirds by pseudoaril) [5]. *Bursera*’s flowers are small and inconspicuous, with few species-specific characteristics, occurring during bursts during the dry season. Thus, in their natural habitats, it is often easier to recognize them by their bark and leaf characteristics as well as the locations where they grow. The *Bursera* genus is closely related to the other two resin producing Burseraceae: *Boswellia* and *Commiphora*, and they differ mostly in their geographic distribution. *Boswellia* and *Commiphora* are present in desert parts of tropical Africa, Arabia, Pakistan, and India, whereas *Bursera* is distributed from the Southern U.S. to Peru and the Caribbean, and particularly in Mexico. The *Bursera* section shares some similarities with *Boswellia*, whereas *Elaphrium* is similar to *Commiphora* [26].

### 2.1. Traditional Uses, Phytochemistry, and Biological Activities

Most of the *Bursera* species that produce lignans are widely used by the Mexican native population. Although different *Bursera* species are used for different health issues, they are traditionally attributed medicinal properties including providing relief from pain, inflammation, rheumatism, and can help treat illnesses such as colds, skin tumors, polyps, and venereal diseases [27,28,29,30]. The following are reported lignan-producing *Bursera* plants from Mexico listed according to the subgenus they belong to and sorted alphabetically. Traditional uses, when found, and biological properties of the isolated compounds are described for each species.

### 2.2. Subgenus Elaphrium

#### 2.2.1. *Bursera citronella* McVaugh and Rzed.

*B. citronella* (synonyms: none reported) is also known as *xochicopal* (Náhuatl name) or *lináloe*, and as *almárciga* in Spanish [31]. It is a 10 m tree with grey trunk bark and unifoliate or trifoliolate leaves, distributed in Western Mexico (Michoacan, Colima, Jalisco, and Guerrero). It belongs to the subsection *glabrifolia* [5]. The resin is mostly used as incense. It has been reported that *B. citronella* is used as antitussive in several regions in Mexico [32].

**Figure 1:**
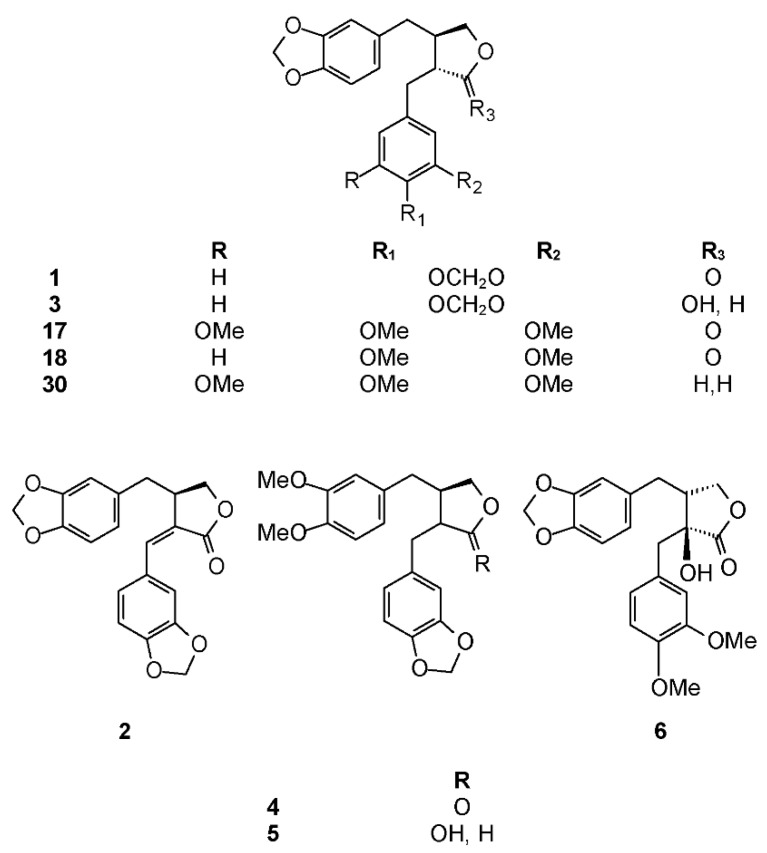
Structures of dibenzyl butyrolactone lignans isolated from *Bursera* spp.

The phytochemistry of *B. citronella* has been studied by Koulman who isolated two lignans: hinokinin (**1**) and savinin (**2**) (Figure 1) [33]. Biological activities of hinokinin have been recently reviewed [34]. Cytotoxicity of hinokinin (**1**) has been tested by several authors [35,36,37,38]. Hinokinin (**1**) has been shown to have anti-inflammatory [39,40,41,42], immunosuppressive [43,44], antibacterial [45], and antiviral [46] properties. Hinokinin (**1**) was tested for several other biological activities, such as antispasmodic [47], neurite outgrowth-promoting in PC12 cells [48], antileukemic [49], antiproliferative [50], and neuroprotective activities [51]. This compound showed an interesting activity against *Trypanosoma cruzi*, [52,53,54,55,56], but with a low parasite selectivity [57]. Hinokinin has been chosen as a trypanosomicidal marker in *P. cubeba* [58]. In order to ascertain the safety of this compound toward mammalian cells, several studies have been performed [59,60,61]. The authors found that hinokinin did not increase DNA damage, demonstrating the absence of mutagenic and genotoxic activities. On the other hand, the results on the antimutagenic potential of this compound showed a strong inhibitory effect against some direct and indirect-acting mutagens.

Savinin (**2**), also called hibalactone [62], was isolated for the first time from *Juniperus* spp. [63]. It has been tested for different biological activities, such as cytotoxicity against several tumor cell lines [36,64,65,66]. Savinin (**2**) was shown to have an anti-inflammatory activity in several assays [40,44,67,68,69], but also interesting antinociceptive, anxiolytic, and antioxidant activities [70,71].

#### 2.2.2. *Bursera cuneata* (Schltdl.) Engl.

*B. cuneata* (synonym: *Elaphrium cuneatum* Schltdl. L.) is a tree that grows up to 10 m in height with no-peeling grey-reddish bark. It has imparipinnate leaves of coriaceous texture with 3 to 13 leaflets, 6.5 cm long and 2.3 cm wide, and margin roughly serrated. Their flowers are clustered in inflorescences up to 8 cm long. Its flowers are white and its fruits are up to 1.2 cm long with a black pit, almost completely covered by a yellow or orange pseudoaril. It is native to Mexican oak-tropical deciduous forest transition zones from Jalisco to Oaxaca and is often known as copal or *copalillo* [72]. Although *B. cuneata* is characterized by seeds covered by pseudoaril, Becerra and Venable did not classify it into the *copallifera* group [22]. No medical uses have been reported for this species, but it is largely used as incense during sacred ceremonies and to prepare handcrafted objects.

Koulman isolated three lignans from this species: hinokinin (**1**), savinin (**2**), and cubebin (**3**) (Figure 1) [33]. Cubebin was first isolated by Chatterjee in 1968 from *Piper cubeba* [73] and then from several *Aristolochia* spp. Biological activities of cubebin have been recently reviewed by Cunha et al. [16]. In particular, trypanocidal activity of this compound against free amastigote forms of *Trypanosoma cruzi* has been studied by de Souza et al. [52], and Bastos et al. showed it is inactive against trypomastigote forms [74]. Notably, cubebin (**3**) is usually the starting material for the semi-synthetic preparation of hinokinin (**1**) and other lignans [52]. Recently, cubebin was proven to induce vasorelaxation via nitric oxide activation without prostacyclin involvement [75]. Because of its therapeutic potential, the effects of cubebin on mutagenicity and genotoxicity has been deeply studied by several research groups [76]. The authors found that cubebin (**3**) was cytotoxic at high doses (280 μM), but at lower concentrations, no cytotoxic, mutagenic, or proliferative effects were observed for this compound. The mutagenicity of cubebin (**3**), alone or in combination with doxorubicin (DXR), using standard (ST) and high bioactivation (HB) crosses of the wing Somatic Mutation And Recombination Test (SMART) in *Drosophila melanogaster* was also studied [77]. Even in this case, the effect of cubebin was dose-dependent. At lower doses (<1 mM), it reduces DXR toxicity, whereas at higher doses (>2.0 mM), it is cytotoxic. The biological activities of cubebin have been recently reviewed [78].

#### 2.2.3. *Bursera excelsa* (Kunth) Engl.

*B. excelsa* (synonyms: *Bullockia sphaerocarpa* and *Elaphrium excelsum*) is commonly known as *tecomajaca*, or *copal santo*, *pom* (in the Maya language) [79] and *tecomahaca* in the Náhuatl language [29]. It belongs to the *copallifera* group. These are trees up to 8 m tall with grey non-peeling bark. Their leaves are 11 to 23 cm long and 6 to 10.5 cm wide, with winged raquis and 9 to 15 leaflets, hairy and margin conspicuously toothed. Their flowers are small, yellow, and densely hairy. Traditionally, it is used to treat tumors and muscle spasms [29]. It is widely distributed across Mexico from the state of Sinaloa to Chiapas. The phytochemistry of *B. excelsa* has been extensively studied [8,12,13]. Regarding lignan composition, Koulman isolated three compounds from this species: 3,4-dimethoxy-3′,4′-methylenedioxylignano-9,9′-lactone (**4**), 3,4-dimethoxy-3′,4′-methylenedioxylignano-9,9′-epoxylignan-9′-ol (DME) (**5**), and guayadequiol (**6**) (Figure 1) [33]. Compound **4** was named iso-bursehernin “…since the only difference between this compound and bursehernin is the placement of the aromatic groups in relation to the lactone ring” and compound **5** was named DME [33]. Iso-bursehernin (also called kusunokinin) was isolated from several plants, such as *Cinnamomun camphora* [80], *Virola* spp. [81,82], and from different species of *Haplophyllum* [83,84,85]. Compounds **4** and **5** were identified as active glioma inhibitors in a bioassay-guided isolation process from *Piper nigrum* fruits [86] and **4** selectively docked to *Leishmania mexicana* pyruvate kinase in a study to find potential antiprotozoal polyphenolic plant extracts [87]. Kusunokinin isolated from *P. nigrum* showed potent cytotoxic activity on breast cancer cells (MCF-7 and MDA-MB-468) with IC_50_ values of 1.18 and 1.62 μg/mL, respectively, but demonstrated lower cytotoxicity on normal breast cell lines (IC_50_ higher than 11 μg/mL). Cell cycle studies showed that this compound induced cell apoptosis and drove cells toward the G2/M phase. Moreover, it decreased topoisomerase II and Bcl-2. The authors observed an increasing in p53, p21, bax, cytochrome c, and caspase-8, -7, and -3 activities, except caspase-9, suggesting that kusunokinin has potent anticancer activity through the extrinsic pathway and G2/M phase arrest [88].

Lignans that contain a methylenedioxy group show high antifeedant or deterrent activity against insects. Polar substituents on the aromatic rings, such as hydroxyl or glycosyl groups, reduce this activity. Guayadequiol (**6**) was isolated for the first time from *Bupleurum salicifolium* [89]. No biological data for this compound have been reported. The hexane extract of *B. excelsa* was shown to possess in vivo anti-inflammatory activity [29].

#### 2.2.4. *Bursera graveolens* (Kunth) Triana and Planch.

**Figure 2:**
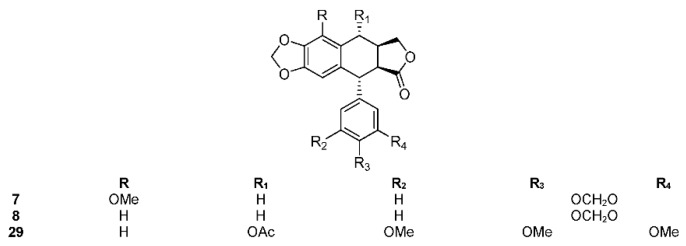
Aryltetraline lignans (*picro* series) isolated from *Bursera* spp.

*B. graveolens* (synonyms: *Amyris caranifera*, *A. graveolens*, *B. andersonii*, *B. pilosa*, *B. tatamaco*, *Elaphrium graveolens*, *E. pilosum*, *Terebinthus graveolens*, and *T. pilosa*) is called copal and mizquixochicopalli in Náhuatl language [90]. Its Spanish common name is *palo santo* and it is native to the tropical dry forests from the Yucatan Peninsula of Mexico, south to Peru, and the Galapagos Islands of Ecuador. These are trees and sometimes shrubs up to 15 m tall, highly fragrant, with grey bark. Their leaves are imparipinnate, sometimes bipinnate, up to 30 cm long and 18 cm wide, with 7 to 11 leaflets. The leaflets are 3 to 9 cm long and 1 to 4 cm wide, of acuminate apex, and margin roughly serrated. Their small flowers are yellowish, white, or green and their fruits glabrous and are up to 1.0 cm long. Their seeds are black and about two-thirds covered by an orange-red pseudoaril. Traditionally, the alcoholic extract of the bark is used for rheumatism, and the bark infusion, as a digestive and for respiratory problems. In recent years, the resin and oils have been extracted from the wood by the perfume industry. From the active methanol extract of stems, Nakanishi et al. isolated a new aryltetralin lignan, burseranin (**7**), and picropolygamain (**8**) (Figure 2) along with known triterpenes, lupeol and epi-lupeol [91]. The two isolated lignans **7** and **8** showed important cytotoxic activity against the human HT1080 fibrosarcoma cell line. Both compounds exhibited potent inhibitory effects in comparison with adriamycin as a positive control (5.5 and 1.9 μg/mL vs. 0.1 μg/mL). Picropolygamain (**8**) was isolated for the first time in 1985 from *Commiphora incisa* resin [92] and later from *Bursera simaruba* [93]. This compound was shown to be active against LNCaP (androgen-sensitive human prostate adenocarcinoma) cell line (ED_50_ 1.1 μg/mL) during tests aimed at developing an in vivo Hollow Fiber Assay [94].

#### 2.2.5. *Bursera penicillata* (Sessé and Moç. ex DC.) Engl.

*B. penicillata* (synonyms: *Amyris penicillata*, *Bursera mexicana*, *Elaphrium delpechianum*, *E. mexicanum*, *E. penicillatum*, *Terebinthus delpechiana*, and *T. mexicana*) belongs to the section *glabrifolia*. Its common names are *coyoluche*, *torote incienso*, and *torote copal* [95]. These are trees up to 12 m tall of grey or reddish grey bark, and are very fragrant, even sometimes from a distance. Their leaves are imparipinnate, 12 to 38 cm long, rachis-winged, and with 3 to 15 leaflets. The leaf blades are finely pubescent on both surfaces and the margins strongly toothed. Their flowers are small, white, and arranged in few to many inflorescences up to 14 cm long. Fruits are 1 to 1.3 cm long, 0.8 to 1.1 cm wide with a black pit, and partially covered by a red, orange, or pale pseudoaril. Endemic to Northwest Mexico, this species prospers in tropical deciduous forests and sporadically thornscrub and transition areas to oak woodland, from Southeastern Sonora and Southwest Chihuahua to Michoacan. According to Gentry, the leaves are used to treat the common cold and the gum is used for toothaches. It is also used as incense [96]. Koulman reported the presence of savinin (**2**) in this species [33].

#### 2.2.6. *Bursera submoniliformis* Engl.

*B. submoniliformis* (synonyms: *Bursera subsessiliformis* Engl. and *Elaphrium submoniliforme* (Engl.) Marchand ex Engl.) is commonly known as *copal chino*. These trees are up to 12 m tall with grey to reddish gray bark. Their leaves are imparipinnate, up to 20 cm long and 7 cm wide, with 9 to 17 leaflets. The leaflets are velvety, 1.3 to 5 cm long, 0.5 to 2 cm wide, and have toothed margins. They have small white flowers that are arranged in inflorescences. Fruits are 7.5 to 12 mm long with a black pit almost or completely covered by a yellow or orange pseudoaril. Endemic to Mexico, this species inhabits tropical deciduous forests at altitudes of 500 to 1600 m of the Balsas and Papaloapan river basins in the states of Mexico, Michoacan, Guerrero, Puebla, Morelos, and Oaxaca. It belongs to the subsection *copallifera*. The gum resin is used to alleviate pain associated with flatulence and tooth-ache [97]. The only reference about the phytochemistry of *B. submoniliformis* is by Koulman, who reported the presence of savinin (**2**) in this species [33].

### 2.3. Section *Bursera*

#### 2.3.1. *Bursera aptera* Ramirez

*B. aptera* (synonyms: *Elaphrium apterum* and *Terebinthus aptera*) belongs to the section *fagaroides.* Its common names are *cuajiote verde* [98,99] and *cuajiote blanco* (Náhuatl names). The species is distributed in Guerrero, Morelos, Oaxaca, and Puebla regions in Mexico [2]. These are shrubs or trees up to 10 m high with green trunks and bark that exfoliates in yellow or beige papyrus-like sheets. The leaves are glabrous, 2.5–7.5 cm long comprising 4 to 9 pairs of leaflets up to 15 mm long and 6 mm wide. The flowers are reddish, yellow, or white and the fruits are small, up to 7 mm long, greyish red when mature, and with a pit completely covered by a yellow or white papery pseudoaril.

**Figure 3:**
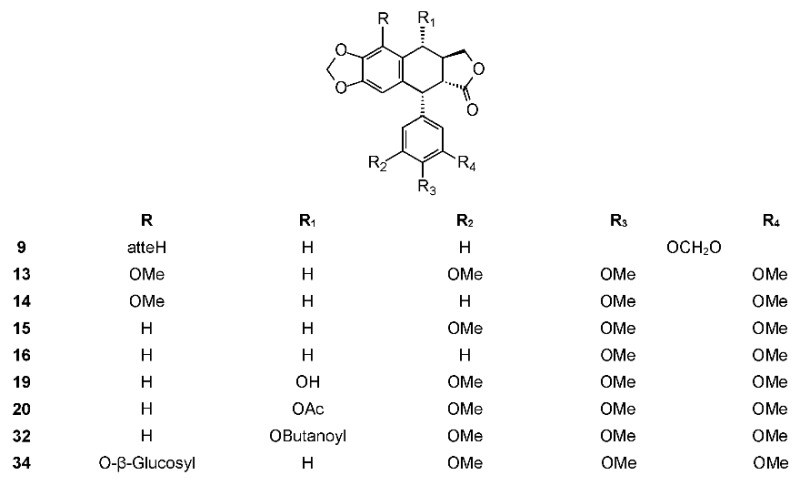
Aryltetraline lignans isolated from *Bursera* spp.

Nieto-Yañez et al. evaluated the anti-leishmanial activity of a *B. aptera* methanolic extract. The extract showed strong activity against *Leishmania mexicana* both in the in vitro and in vivo tests. The gas chromatography-mass spectrometry (GC-MS) phytochemical analysis of the extract showed the presence of 11 compounds. Most of these compounds were fatty acids and fatty acid esters, but they revealed the presence of podophyllotoxin (**19**) (Figure 3) [100].

#### 2.3.2. *Bursera arida* (Rose) Standl.

*B. arida* (synonyms: *Elaphrium aridum* Rose and *Terebinthus arida* Rose) is endemic in the states of Oaxaca and Puebla [2]. It is commonly known as *zapotillo* [99]. These are small trees, often shrubs, of reddish-brown exfoliating bark with leaves up to 2.5 cm long and 1 cm wide, comprising 3 to 11 leaflets. Their flowers are very small, reddish, and solitary. Their fruits are solitary or in pairs, over short and pilose peduncles 1 to 2 mm long, with a seed completely covered by a pale yellow pseudoaril [101]. It belongs to the *microphylla* subsection. Traditionally, the plant latex is topically used for healing wounds and skin eruptions [102] in the Tehuacán-Cuicatlán valley. *Bursera arida* has different medicinal uses such as a disinfectant, cough suppressant, and antidepressant [103].

**Figure 4:**
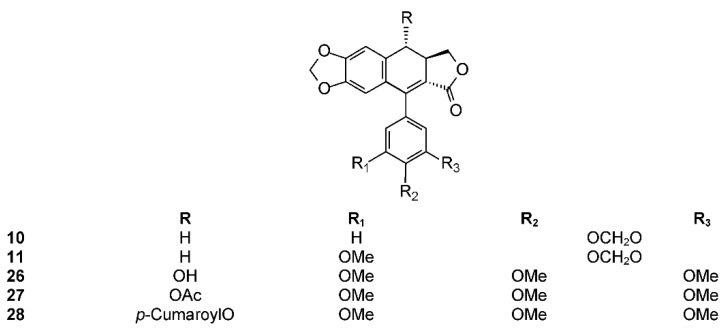
7′,8′-Dehydro-aryltetraline lignans isolated from *Bursera* spp.

**Figure 5:**
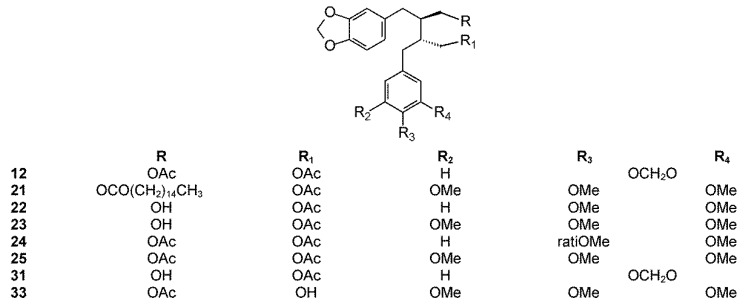
Dibenzylbutane diol lignans isolated from *Bursera* spp.

The phytochemistry of *B. arida* was studied by Ionescu, who prepared a chloroform extract of the stems, leaves, twigs, and bark, and found naringenin, β-sitosterol, betulonic acid, and four lignans: (+)-3-hydroxymethyl-5-methoxy-6,7-methylenedioxy-1-(3′,4′-methylenedioxybenzene)-l,2,3,4-tetrahydronaphthalene-2-carboxylic acid lactone (**9**) (Figure 3), (+)-3-hydroxymethyl-6,7-methylenedioxy-1-(3′,4′-methylenedioxybenzene)-3,4-dihydronaphthalene-2-carboxylic acid lactone (**10**) (Figure 4), (+)-3-hydroxymethy1-6,7-methylenedioxy-1-(5′-methoxy-3′,4′-methylenedioxybenzene)-3,4-dihydronaphthalene-2-carboxylic acid lactone (**11**) (Figure 4), and 2,3-bis-(3,4-methylenedioxybenzyl)butane-l,4-diol diacetate (**12**) (Figure 5) [104]. Compound **12** has the structure of ariensin.

#### 2.3.3. *Bursera ariensis* (Kunth) McVaugh and Rzed.

*B. ariensis* (synonyms *B. panosa* Engl., *B. sessiflora* Engl., *E. ariensis* Kunth, and *E. brachypodium* Rose). These are trees and sometimes shrubs between 2 and 8 m tall with greenish-gray trunks and bark that exfoliates in papery sheets that are yellowish or beige, sometimes with orange tones, with whitish resin that darkens upon contact with air. Their leaves are hairy especially when young, 5 to 22 cm long, and 2 to 7 cm wide, with a winged rachis and 5 to 9 leaflet pairs. Their flowers can be solitary but often develop in conglomerates at the end of branches of reddish-yellow color. Their fruits are 6 to 8 mm long, growing in thick conglomerates, with a pit completely covered by a yellow or orange pseudoaril. It is distributed in Mexico (Chiapas, Guerrero, Jalisco, and Oaxaca regions) where it is commonly known as *guande* and *cuajiote blanco* (Náhuatl name) [101] and used to treat colds and inflammation. It belongs to the *fagaroides* group and is poorly studied. From the acetonic extract of the bark, Hernandez isolated a new lignan named ariensin (**12**) [105]. Ariensin was shown to be active against the RAW246.7 murine cell line (IC_50_ 9.8 μM) [11].

#### 2.3.4. *Bursera fagaroides* (H.B.K.) Engl.

*B. fagaroides*, or “fragrant bursera” (*B. obovata* Turcz., *B. schaffneri* S. Wats) [2] is a dioecious shrub or tree, occasionally hermaphrodite, 0.5 to 10 m high, and highly resinous. The trunk is green with a bark that exfoliates in yellowish-gray papery sheets. The leaves are most often compound, with 5 to 13 leaflets, although occasionally they are unifoliolate or trifoliolate. They have whitish-green or yellow flowers, with a few of them arranged in small inflorescences or solitary. Male flowers are most often 5-merous (sometimes 3- and 4-). Female flowers are 3-merous. Fruits are typically 0.5 to 0.8 cm long with short peduncles no more than 2 mm long that terminate in a sharp point. When the pits mature, they are covered by a yellow or red pseudoaril. It is commonly known as *cuajiote amarillo* (in Morelos) or *pima bajo* [106]. This species belongs to the *fagaroides* group. Three subspecific variants of this species were recognized by McVaugh and Rzedowski (1965). *B. fagaroides* var. *fagaroides*, commonly known as *xixote* and *jiote* [72], are most often shrubs with leaves with serrated margins and are distributed in Northern, Central, and Western Mexico. *B. fagaroides* var. *purpusii* (common name *aceitillo*) [107] are most often trees with leaves of entire margin (not toothed) distributed throughout Southeastern Mexico. *B. fagaroides* var. *elongata,* common Mayo name *to’oro sahuali* [95], is from Northwestern Mexico, and has been classified by recent molecular studies as a separate taxon not belonging within *B. fagaoides* [108].

*Bursera fagaroides* is used to alleviate inflammation, skin tumors, and warts, and is perhaps the most studied *Bursera* species in terms of its chemistry and biological effects. The first report about *B. fagaroides* phytochemistry dates back to 1969, when Bianchi et al. isolated β-peltatin A-methyl ether (**13**) and the new 5′-demethoxy-β-peltatin A-methyl ether (**14**) (Figure 3) that showed activity against the Walker carcinoma 256 (WA16) tumor system from the chloroform extract of this plant. The ethanol extract from the dried exudate of *Bursera fagaroides* showed significant cytotoxic activity against the HT-29 (human colon adenocarcinoma) cell line. From this extract, Velazquez-Jimenez et al. isolated two aryltetraline lignans: (−)-deoxypodophyllotoxin (**15**) and (−)-morelensin (**16**) (Figure 3), and two dibenzylbutirolactone lignans: (−)-yatein (**17**) and (−)-5′-desmethoxy yatein (**18**) or bursehernin] (Figure 1) [109]. The authors determined the absolute configuration of these compounds by comparing the vibrational circular dichroism spectra of known podophyllotoxin and deoxypodophyllotoxin with those obtained by density functional theory calculations. Morelensin (**16**) was shown to be cytotoxic and antiproliferative against several cancer cell lines [35,110]. Yatein (**17**) was shown to be cytotoxic and antiproliferative to several cancer cell lines [111,112,113,114]. For example, it was tested against HL-60 (human promyelocytic leukemia cells), SMMC-7721 (human hepatoma), A-549 (human lung adenocarcinoma), MCF-7 (human breast cancer), and SW480 (human colon adenocarcinoma) cell lines using the MTT (3-(4,5-dimethylthiazol-2)2,5-difeniltetrazolium bromide) method as previously reported [115], with cisplatin as the positive control [116]. Yatein (**17**) showed significant cytotoxic activity against all tested cell lines being superior to cisplatin. Chen et al. tested yatein [117] against DLD-1 (human colorectal carcinoma), CCRF-CEM (human lymphoblastic leukemia), and IMR-32 (human neuroblastoma) cell lines. The study showed that yatein possesses similar cytotoxic activity to doxorubicin (positive control) against DLD-1 and CCRF-CEM cell lines [117]. Other studies showed that it is able to suppress herpes simplex virus type 1 (HSV-1) replication in HeLa cells in a plaque reduction assay. Doussot et al. studied the lignan profile and the antiproliferative activity of ethanol extracts from plants belonging to different species of *Linum*, *Callitris*, and *Juniperus*. They compared the activity of deoxypodophyllotoxin (**15**), podophyllotoxin (**19**), and yatein (**17**) against six human cancer cell lines: A549, U373 (glioblastoma), T98G (glioma), Hs683 (oligodendro-glioma), MCF7 (breast cancer), and SKMEL-28 (melanoma). The most active compound was deoxypodophyllotoxin (IC_50_ < 0.01 μM against all cell lines, not tested against U373), followed by podophyllotoxin (IC_50_ = 0.03 μM against all cell lines except U373 (IC_50_ > 100 μM). Yatein (**17**) showed an antiproliferative activity, but to a lesser extent (IC_50_ = 30.9, not tested, 26.5, 29.8, 31.9, and 39.6 μM, respectively) [118]. The inhibitory effect of yatein (**17**) on HSV-1 replication was concentration-dependent with an IC_50_ value of 30.6 ± 5.5 μM [119]. Furthermore, yatein (**17**) was demonstrated to be a potent CYP3A4 inhibitor and this study is of particular importance as **17** and other methylenedioxyphenol compounds were found to induce herb-drug interactions in clinical situations [120]. Yatein (**17**) showed other important biological activities, such as anti-platelet aggregation [121], and was shown to have moderate inhibitory activity against cytochrome P450 [122]. The bioactivity-guided separation of the hydroalcoholic extract of *B. fagaroides* var. *fagaroides* by Rojas-Sepúlveda et al. led to, besides the already isolated lignans, podophyllotoxin (**19**), burseranin (**7**), and acetyl podophyllotoxin (**20**) (Figure 3). All the isolated compounds were found to be active against tumor cell lines tested, especially 5′-demethoxy-β-peltatin A-methyl ether (**14**), which exhibited greater activity than camptothecin and podophyllotoxin against PC-3 (ED_50_ = 1.0 × 10^−5^ μg/mL) and KB (ED_50_ = 1.0 × 10^−5^ μg/mL) cell lines [123]. Furthermore, the cytotoxic and antitumor activity of the ethanol extract (70%) of *B. fagaroides* bark against L5178Y lymphoma cell line was tested. The antitumor activity was studied on BALB/c mice (2 × 10^4^ cells L5178Y i.p). Treated animals (at 50 mg/kg/day over 15 days) showed a significant increase in survival compared with those treated with the placebo or without treatment [124].

Podophyllotoxin and deoxypodophyllotoxin are secondary metabolites of many plants [125]. The biological activities and the importance of podophyllotoxin (**19**), as the lead compound in the development of new anticancer agents, are well known [126,127,128]. The problem connected with its use is the scarce amount isolated from natural sources. For this reason, biotechnological production of this lignan has been studied [129]. Deoxypodophyllotoxin (**15**) is a promising anticancer agent [130,131].

Other diarylbutane lignans were isolated by Morales-Serna et al. from the chloroform extract of *B. fagaroides* resin, named by the author as 9′-acetyl-9-pentadecanoyl-dihydroclusin (**21**) (correctly, a hexadecanoyl derivative), 2,3-demethoxy-secoisolintetralin monoacetate (**22**), and dihydroclusin monoacetate (**23**), together with two known lignans: 2,3-demethoxy-secoisolintetralin diacetate (**24**) and dihydroclusin diacetate (**25**) [132] (Figure 5). Recently, Antúnez Mojica et al. isolated three new aryldihydronaphtalene-type lignans from the dichloromethane stem bark extract of *B. fagaroides* var. *fagaroides*: 7′,8′-dehydropodophyllotoxin (**26**), 7′,8′-dehydroacetylpodophyllotoxin (**27**), and 7′,8′-dehydro-*trans*-p-cumaroyl podophyllotoxin (**28**) (Figure 4), along with six known lignans: podophyllotoxin (**19**), acetylpodophyllotoxin (**20**), 5′-demethoxy-β-peltatin A methylether (**14**), acetylpicropodophyllotoxin (**29**) (Figure 2), burseranin (**7**), and hinokinin (**1**) [133]. The cytotoxic activity of the new isolated compounds **26**–**28** against the cancer cell lines KB, PC-3, MCF-7, and HF-6 was evaluated, which showed that all of them displayed good activity against KB, PC-3, and HF-6, but were not active against the MCF-7 cell line. When compared with podophyllotoxin (**19**) (ED_50_ = 2.10 × 10^−4^ μM), compounds **26** and **28** were most active against the PC-3 cell line displaying similar toxicity (ED_50_ = 2.4 × 10^−5^ and 2.42 × 10^−5^ μM, respectively), whereas compound **27** was less active (ED_50_ = 0.06 μM). Lignans **26**–**28** showed moderate activity against KB and HF-6 cell lines when compared to **19**. The cytotoxic activity of the other isolated compounds was already proven [123]. Acetylpicropodophyllotoxin (**29**) was previously isolated from *Hernandia ovigera* and it is a potent, selective inhibitor of type I insulin-like growth factor receptor (IGF-IR) [134].

Ornithine decarboxylases (ODC) are enzymes that catalyze the decarboxylation of ornithine to produce putrescine in the biosynthesis of polyamines. Polyamine metabolism is closely related with the progression of growth, proliferation, and cell regeneration. The in vitro effect of an ethanolic extract from the stem bark of *Bursera fagaroides* on ODC activity, and on the growth of *Entamoeba histolytica*, was studied by Rosas-Arreguín et al. using metronidazole and G418 as positive controls [135]. The authors found growth inhibition, with IC_50_ values in the order of 0.05 mg/mL. The ODC activity was inhibited by 12% at 4.0 mg/mL.

Gutiérrez-Gutiérrez et al., considering the use in Mexican traditional medicine of *B. fagaroides* as an antidiarrheic, investigated the in vitro anti-giardial activities of four podophyllotoxin-type lignans from *Bursera fagaroides* var. *fagaroides*: 5′-demethoxy-β-peltatin A methylether (**14**), acetylpodophyllotoxin (**20**), burseranin (**7**), and podophyllotoxin (**19**) [136]. They found that all lignans affected *Giardia* adhesion, but only compounds **14**, **19**, and **20** caused growth inhibition.

#### 2.3.5. *Bursera microphylla* A. Gray

*B. microphylla*, or “elephant tree” (*Elaphrium microphyllum* (A. Gray) Rose, *Terebinthus microphylla* (A. Gray) Rose), is commonly known as *xoop* (Seri name) and *torote blanco* [95]. It is typically a small tree that grows up to 10 m tall, with a thickened trunk and thickened lower branches, with light gray to white peeling bark, with younger branches having a reddish color. The leaves are 3 to 8 cm long and have 7 to 35 small, linear, and glabrous leaflets. The leaflets are up to 1.5 cm long. The flowers are yellowish white or greenish and inconspicuous. The fruits are brownish red at maturity with a black pit completely covered by a yellow-orange pseudoaril [26]. It belongs to the *microphylla* group. The Seri Indians from Sonora, Mexico use the bark, leaves, flowers, and fruits to treat a variety of maladies such as inflammation, diarrhea, and venereal diseases. The first lignan isolated from *Bursera microphylla* was burseran (**30**) (Figure 1) [137]. Cole et al. showed that burseran has cytotoxic activity against human epidermoid carcinoma of the nasopharynx (9KB cell line) in a Cancer Chemotherapy National Service Center (CCNSC) test (ED_50_ < 10 μg/mL) acting as a spindle poison. Tomioka et al. synthetized *cis* (H_8_′ α) and *trans* burseran and tested them in a cilia regeneration test in *Tetrahymena*, that is a useful model for studying the antitubulinic activity of spindle poisons [138]. Both *trans* and *cis* burseran have shown inhibitory activity, but the antitumor activity was higher for *trans* burseran [139]. An analysis of the chemical composition of the methanol extract of *B. microphylla* resin from the Sonora Desert (Mexico), revealed the presence of several known lignans: ariensin (**12**), burseranin (**7**), dihydroclusin diacetate (**25**), picropolygamain (**8**), desmethoxy-yatein (**18**), hemiariensin (**31**) (Figure 5), and dihydroclusin 9′-acetate (**23**); and two new ones: podophyllotoxin butanoate (**32**) and dihydroclusin 9-acetate (**33**) (Figure 3 and Figure 5) [11,140]; in addition to burseran (**30**). Compound **32** was already known as a synthetic derivative of podophyllotoxin [141] but it was new as a natural product. Burseran (**30**) and dihydroclusin diacetate (**25**) were tested against human cancer cell lines: A549 (lung cancer), HeLa (cervix cancer), and PC-3 (prostate cancer), and on murine cell lines M12.C3.F6 (B cell lymphoma) and RAW264.7 (macrophages transformed by virus Abelson leukemia); which were found to be more active against murine cell lines in micromolar range (IC_50_ 13.8, 36.3, and 2.5 μM, respectively). The anti-proliferative activities of dihydroclusin 9-acetate (**31**), dihydroclusin 9′-acetate (**23**), burseranin (**7**), picropolygamain (**8**), and hemiariensin (**31**) were evaluated on the human cancer cell lines A549, LS 180, and HeLa, and on the human non-cancer cell line ARPE-19. None of the evaluated compounds had statistically significant anti-proliferative effects with respect to dimethyl sulfoxide (DMSO) control on LS 180, A549, and ARPE cell lines. However, burseranin (**7**) and picropolygamain (**8**) had an interesting anti-proliferative activity on the gynecological cancer cell line, HeLa, with IC_50_ values of 21.72 ± 1.03 and 9.31 ± 1.01 µM, respectively [140].

#### 2.3.6. *Bursera morelensis* Ramírez

*B. morelensis* (synonym: *Elaphrium morelense* (Ramírez) Rose) is widely distributed in Mexico [101], where it is commonly known as *coabinillo* [99]. It is a tree up to 13 m tall with red bark that exfoliates in thin sheets. Its leaves are 5 to 11 cm long and 1.5 to 4.5 cm wide, with 15 to 51 linear leaflets. The flowers are yellow, pink, greenish, or white. The fruits are 0.5 to 1 cm long, with a pit completely covered by a pale yellow pseudoaril. It belongs to the *microphylla* group. The only two papers describing the phytochemistry of *B. morelensis* address the composition and the anti-inflammatory activity of the essential oil [142] and the isolation of deoxypodophyllotoxin (**15**) and morelensin (**16**) from the resin [110]. Morelensin (**16**), although highly active against the KB (epidermoid carcinoma) cancer cells, demonstrated only marginal activity against the porcine stable (PS) kidney cell line [110].

#### 2.3.7. *Bursera roseana* Rzed., Calderón & Medina

*B. roseana* (synonyms: *Bursera acuminata* (Rose) Engl. and *Terebinthus acuminata* Rose) is a 12–20 m high tree with bark peeling in reddish-orange stripes. It is imparipinnate with 3 to 7 (sometimes 9) leaflets with a hairy underside but a bright and glabrous upper side. The leaflets are typically of oval shape, 4.5 to 15 cm long and 2 to 6 cm wide, ending in a long point. The flowers are white or greenish and the is fruit is glabrous, 0.9 to 1.2 cm long, with a pit completely covered by a pale pseudoaril [143].

This species grows in moist canyons in the transition zone between highland pine-oak forest and lowland tropical subdeciduous forest. It is common in Nayarit, Zacatecas, Aguascalientes, Jalisco, Colima, Michoacán, Estado de México, and Guerrero [143]. It belongs to the *simaruba* group. The only phytochemical study on this species was reported by Koulman, who found 5′-desmethoxy yatein [bursehernin or (−)-*trans*-methylpluviatolide [144] (+)-*trans*-methylpluviatolide, which has been called dextrobursehernin [145] (**18**)], morelensin (**16**), deoxypodophyllotoxin (**15**), and β-peltatin-A methylether (**13**). Bursehernin (**18**) was studied by Ito et al. for its inhibitory effects on Epstein-Barr virus early antigen activation induced by 12-*O*-tetradecanoylphorbol 13-acetate in Raji cells [146]. The data reported demonstrated that bursehernin (**18**) was slightly weaker than β-carotene, which is commonly used in cancer prevention studies [147] so it might be a valuable antitumor promoter. Bursehernin (**18**) is able to inhibit the growth of *Neisseria gonorrhoeae* [148]. The trypanocidal activity of racemic mixtures of *cis*- and *trans*-bursehernin was evaluated in vitro against trypomastigote forms of two strains of *Trypanosoma cruzi*, and results showed that the racemic *cis*-stereoisomer was inactive, whereas the racemic *trans*-stereoisomer displayed trypanocidal activity, with an IC_50_ ~89.3 μM. These results were different from those obtained for pure (−)-*trans*-methylpluviatolide by Bastos et al., but the difference could be ascribed to the use of the racemic mixture [74].

#### 2.3.8. *Bursera schlechtendalii* Engl.

*B. schlechtendalii* (synonyms: *Bursera jonesii* Rose, *Elaphrium jonesii* (Rose) Rose, *Terebinthus jonesii* (Rose) Rose, and *Terebinthus schlechtendalii* (Engl.) Rose) is a small tree or shrub, 4–6 m high, with a strong turpentine smell, and known as *sak chakaj*. It has a glossy greyish pink bark that peels off in thin papery sheets; the branches are thick and stout. The leaves are simple (unifoliolate), often less than 6 cm long and 2.5 cm wide. The flowers are small, usually solitary, with yellow or reddish petals, and the fruits are 4 to 8 mm long with a pit completely covered by a yellow or red pseudoaril. It is used to treat the flu. It is found at altitudes of 200–400 m on dry rocky hillsides or in thickets in Southern Mexico and Guatemala. It belongs to the *fagaroides* group. In 1972, McDoniel et al. isolated from stems and leaves the chloroform extract of Mexican *B. schlechtendalii* Engl. yatein (**17**) and bursehernin (**18**).

#### 2.3.9. *Bursera simaruba* (L.) Sarg.

*B. simaruba* or “*gumbo-limbo*” (*Bursera simaruba* var. *yucatanensis* Lundell) is commonly known as *yala-guito* [99]. It is a 6–15 m high tree with a peeling reddish bark that reveals a smooth grey underbark. The leaves are compound, bright, and mostly glabrous when mature. Leaves have 3 to 13 leaflets, 4 to 9 cm long and 1.8 to 3.5 cm wide. The small flowers have pink, pale yellow-green, or white petals and are arranged in inflorescences. The fruits are glabrous, red to brownish, 1 to 1.5 cm long, ending in a point, with a pit completely covered by a red pseudoaril. This is perhaps the most widespread species of *Bursera*, occurring from Southern Florida and the Caribbean, along both coasts of Mexico, to South America. Taken orally or as curative baths, the leaves and bark are attributed a variety of medicinal properties. It belongs to the *simaruba* group. In 1992, Peraza-Sánchez and Peña-Rodriguez isolated picropolygamain (**8**), which showed activity in the brine shrimp assay (LC_50_ = 52.2 ppm). Further in vitro evaluation against three human tumor cell lines (A-549, lung), MCF-7 (breast), and HT-29 (colon) showed that **8** has cytotoxic activity comparable to that of Adriamycin [93]. Noguera et al. isolated the anti-inflammatory β-peltatin A-methyl ether (**13**) from the leaf hexane extract of *B. simaruba.* It inhibited the carrageenan-induced rat paw edema, in a dose- and time-dependent manner (three hours = 9.55%, five hours = 34.37%, and seven hours = 35.6%) [149]. Maldini et al. studied the methanolic extract of *Bursera simaruba* bark and isolated 11 compounds, including lignans yatein (**17**), β-peltatin-*O*-β-d-glucopyranoside (**34**) (Figure 3), hinokinin (**1**), and bursehernin (**18**) [149].

## 3. Conclusions

Lignans are phenolic secondary metabolites characterized by a large variety of biological activities. Among these, the cytotoxic and anti-proliferative ones are perhaps the most common and most studied. Literature analysis of Mexican plants producing lignans *Burseara* spp., revealed that the most common lignan types are dibenzyl butyrolactones (nine compounds), picro aryltetraline derivatives (three compounds), aryltetraline derivatives (nine compounds), 7′,8′-dehydro-aryltetraline derivatives (five compounds), and dibenzylbutane diols (eight compounds). Notably, all the examined *Elaphrium* subgenus species produce only dibenzyl butyrolactones and picro aryltetraline derivatives, whereas *Bursera* subgenus produce all lignan types. The most common compound, up to now, appears to be bursehernin (**18**), which is present in five species belonging to the *Bursera* section. Hinokinin (**1**) and savinin (**2**) are also widespread (four species), both in the *Elaphrium* and *Bursera* sections. Compounds **3**, **8**, **12**, **13**, **15**, **16**, **17**, and **33** were isolated from three species.
